# High recent PrEP adherence with point‐of‐care urine tenofovir testing and adherence counselling among young African women: results from the INSIGHT cohort

**DOI:** 10.1002/jia2.26389

**Published:** 2024-12-09

**Authors:** Brenda Gati Mirembe, Deborah Donnell, Meighan Krows, Zinhle Zwane, Elizabeth Bukusi, Ravindre Panchia, Cheryl Louw, Noluthando Mwelase, Pearl Selepe, Melissa Senne, Logashvari Naidoo, Rachel Chihana, Margaret Kasaro, Harriet Nuwagaba‐Biribonwoha, Philip Kotze, Katherine Gill, Pippa MacDonald, Alastair vanHeerden, Shannon Bosman, Manjeetha Jaggernath, Phillip du Preez, Amy Ward, Remco P. H. Peters, Sinead Delany‐Moretlwe, Sue Peacock, Rachel Johnson, Jason Caucutt, Susan Morrison, Guohong Wang, Monica Gandhi, Jennifer Velloza, Renee Heffron, Connie Celum, the INSIGHT Study Team

**Affiliations:** ^1^ Makerere University‐Johns Hopkins University Kampala Uganda; ^2^ Fred Hutchinson Cancer Center Seattle Washington USA; ^3^ Department of Global Health University of Washington Seattle Washington USA; ^4^ Setshaba Research Centre Pretoria South Africa; ^5^ Kenya Medical Research Institute Nairobi Kenya; ^6^ Perinatal Research HIV Research Unit Soweto South Africa; ^7^ Madibeng Centre for Research Madibeng South Africa Brits South Africa; ^8^ Helen Joseph Hospital Clinical HIV Research Unit University of the Witwatersrand Johannesburg South Africa; ^9^ Aurum Klerksdorp Clinical Research Centre Klerksdorp South Africa; ^10^ Aurum Rustenburg Clinical Research Centre Rustenburg South Africa; ^11^ South African Medical Research Center Chatsworth Clinical Research Site Chatsworth South Africa; ^12^ Kamuzu University of Health Sciences – Johns Hopkins Research Project Blantyre Malawi; ^13^ University of North Carolina Global Projects Zambia Lusaka Zambia; ^14^ International Center for AIDS and Treatment Program Mbabane eSwatini; ^15^ Qhakaza Mbokodo Research Clinic Ladysmith South Africa; ^16^ Desmond Tutu HIV Centre University of Cape Town Cape Town South Africa; ^17^ Human Sciences Research Council Center for Community Based Research (CCBR) Pietermaritzburg South Africa; ^18^ MATCH Research Unit University of the Witwatersrand Durban South Africa; ^19^ Department of Medicine University of Cape Town, Vuka Research Clinic Cape Town South Africa; ^20^ Foundation for Professional Development Pretoria South Africa; ^21^ Wits Reproductive Health and HIV Unit University of the Witwatersrand Johannesburg South Africa; ^22^ Abottt Research Laboratories Pomona California USA; ^23^ University of California San Franscisco San Fransisco California USA; ^24^ Department of Medicine University of Alabama at Birmingham Birmingham Alabama USA; ^25^ Departments of Global Health Medicine, and Epidemiology, University of Washington Seattle Washington USA

**Keywords:** adherence, Africa, point‐of‐care, PrEP, tenofovir, women

## Abstract

**Introduction:**

Adolescent girls and young women (AGYW) account for two‐thirds of new HIV infections in Africa. African AGYW have had high uptake of oral HIV pre‐exposure prophylaxis (PrEP) but low adherence, which might be improved by point‐of‐care adherence monitoring with tailored counselling.

**Methods:**

From August 2022 to July 2023, we conducted a PrEP demonstration project with sexually active AGYW ages 16−30 years from 20 sites in South Africa, Eswatini, Kenya, Malawi, Uganda and Zambia. Participants were offered oral tenofovir‐based PrEP at enrolment and followed up at 1, 3 and 6 months. PrEP adherence was assessed by a point‐of‐care qualitative lateral flow urine tenofovir (TFV) assay indicating PrEP use in the prior 4 days, which accompanied real‐time adherence counselling that incorporated urine TFV results when testing was available (70.8% of month 1, 35.3% of month 3 and 83.9% of month 6 visits). We estimated overall adherence, correcting for missing test results, and analysed the association of having received urine TFV results at month 1 or 3 with subsequent urine TFV test positivity, using modified Poisson regression.

**Results:**

Of the 3087 AGYW enrolled, the median age was 24 years (interquartile range 21−27), 75.7% were from South Africa, 2878 (93.2%) initiated PrEP at enrolment and 107 (3.5%) after enrolment. Visit retention was 92.0−96.2% for months 1, 3 and 6, and 2518 (90.1%) exited the study with a PrEP refill. Adherence, based on the point‐of‐care urine tenofovir test positivity rate, was estimated as 72%, 71% and 65% at months 1, 3 and 6, respectively. Women who received one prior urine TFV test had a 42% higher likelihood of a subsequent positive urine TFV test (adjusted odds ratio, OR = 1.42, 95% confidence interval, CI 1.27−1.60), and those having received two prior tests had a 67% higher likelihood (adjusted OR = 1.67; 95% CI 1.41−1.98). Observed HIV incidence was 1.38/100 person‐years (95% CI 0.97−2.08).

**Conclusions:**

Oral PrEP uptake, recent adherence and persistence were high in a multisite cohort of young African women over 6 months of follow‐up. The use of a novel point‐of‐care tenofovir assay with tailored real‐time adherence counselling was associated with increased adherence to PrEP at subsequent visits, warranting further study.

**Clinical trials registration:**

clinicaltrials.gov NCT05746065

## 1 INTRODUCTION

While HIV incidence is declining globally, adolescent girls and young women (AGYW) in eastern and southern Africa have shown slower declines, accounting for 63% of new HIV acquisitions in Africa in 2021 [1]. High coverage and effective use of HIV pre‐exposure prophylaxis (PrEP) is a cornerstone of substantially reducing new HIV acquisitions among African AGYW.

In the decade since the first regulatory approval of tenofovir disoproxil fumarate/emtricitabne (TDF/FTC) as PrEP in 2012 and WHO recommendations for PrEP for persons at substantial HIV risk in 2015, demonstration projects among African AGYW have shown that initial oral PrEP uptake is high but that adherence declines and a substantial proportion discontinue within the first 6−12 months [2]. Encouragingly, a pooled analysis of 11 PrEP demonstration projects conducted globally among cisgender women high levels of protection and similar reductions in HIV incidence reduction among women taking four to six doses per week as the recommended seven doses per week [3].

To achieve higher adherence and greater HIV prevention benefits from PrEP, AGYW may benefit from adherence support strategies, including counselling that is targeted with drug‐level feedback using objective measures of adherence (i.e. measuring tenofovir or tenofovir metabolite levels in plasma, dried blood spots, peripheral mononuclear cells or hair) [4, 5, 6]. However, few laboratories perform this testing and the turnaround time for results is typically 1−3 months, creating barriers to real‐time use of these metrics [4]. Point‐of‐care (POC) assays for adherence offer new advantages. The potential benefits of real‐time feedback have been demonstrated for HIV treatment, in which counselling based on POC viral loads was associated with higher rates of viral suppression in South Africa [7, 8]. A lateral flow antibody‐based assay to detect urine tenofovir (TFV) ingestion within the past 4 days has been developed and validated, which provides results in 3−5 minutes [9, 10, 11, 12]. This POC urine TFV assay has been shown retrospectively to correlate with protection from HIV seroconversion in PrEP cohorts [13, 14], to increase adherence prospectively in a cohort of young Kenyan women [15], to improve accuracy of self‐reported PrEP adherence among women in Uganda [16] and to increase HIV viral suppression prospectively among non‐virally suppressed people living with HIV in Namibia [11].

To further evaluate the use of this novel POC urine TFV assay to assess and support PrEP adherence among African AGYW, we evaluated adherence rates using the assay. We also assessed whether the urine TFV test and real‐time adherence counselling affected subsequent PrEP adherence in a multisite cohort of African AGYW initiating PrEP.

## 2 METHODS

### 2.1 Study design and participant recruitment

The INSIGHT study was a prospective, open‐label cohort study which assessed uptake and adherence to daily oral PrEP with TDF/FTC among young African women ages 16−30 years. The study was implemented from August 2022 to July 2023 in 20 research clinics in six countries (15 in South Africa, one each in each in Eswatini, Kenya, Malawi, Uganda and Zambia) (NCT05746065). Participant recruitment was conducted through community sensitization and outreach activities, snowball recruitment and participant‐based referrals, social media, and from a waiting list of possible participants for a truncated efficacy trial of once‐monthly oral islatravir for HIV prevention. At enrolment, women were offered HIV testing per the national algorithm, contraception and PrEP. Women were eligible for the INSIGHT cohort if they were willing to provide written informed consent, tested HIV negative at screening, had vaginal sex in the past 3 months and were interested in PrEP. A plan to initiate PrEP was not a requirement for enrolment. Participants with reactive HIV tests at screening were referred to HIV treatment centres for further management and excluded from the cohort study [17].

### 2.2 Study procedures

At enrolment, interviewer‐administered questions were administered via electronic case report forms (DF Explore, DFNet, Seattle, Washington) to obtain information on demographic characteristics, sexual behaviour, contraceptive use, fertility intentions, clinical history, HIV risk perception, and to calculate the modified VOICE HIV risk score (maximum possible risk score of eight) with higher scores associated with higher risk of HIV acquisition [18].

Questionnaires assessing sexual behaviour, contraceptive use, experiences with PrEP and PrEP adherence were administered at follow‐up visits, which were scheduled at 1, 3 and 6 months after enrolment. Visit reminders were conducted through phone calls and text messages. At enrolment and follow‐up visits, participants were counselled about HIV prevention and given the option to initiate or continue PrEP. Participants were counselled that they could discontinue using PrEP and resume if they needed it during the study. Counsellors asked about barriers and facilitators to PrEP use at each visit. At the end of the study, participants were offered a refill to continue using PrEP and referred to public facilities for continued access to PrEP. Participants were compensated for each study visit attended based on local and national guidelines (e.g. 150 South African Rand equivalent to approximately $15).

### 2.3 Laboratory procedures

HIV testing was performed on‐site at all visits following national guidelines which include serial or parallel rapid HIV tests and confirmation of reactive rapid tests by a third‐ or fourth‐generation antigen/antibody assay. For participants who desired PrEP, same‐day PrEP starts were implemented. Samples were drawn for hepatitis B surface antigen and creatinine testing with results provided as soon as available in alignment with the standard of care. TDF/FTC‐based PrEP was withheld for participants who had an estimated creatinine clearance <60 ml/minute, calculated using the Cockcroft‐Gault equation. Urine pregnancy testing was performed when clinically indicated or requested by the participant. Women could continue using PrEP during pregnancy and breastfeeding, following national guidelines for PrEP in each study country.

### 2.4 POC urine tenofovir test

At the month 1, 3 and 6 follow‐up visits, a POC lateral flow antibody‐based immunoassay for detection of TFV in urine was conducted to provide a qualitative measure of tenofovir adherence (Abbott Rapid Diagnostics, Pomona, CA) for participants who had received PrEP at the prior visit. The assay uses a threshold of >1500 ng/ml for positivity, indicating tenofovir ingestion in the prior 4 days, and in validation testing has demonstrated high sensitivity (100%) and specificity (95%) relative to the gold standard liquid chromatography tandem‐mass spectrometry method [10, 11, 12, 14, 19]. Study staff recorded results in an electronic case report form and uploaded an image of each test strip to enable data quality checks by a central data quality manager at the University of Washington. After the study launch, delays in procurement and importation of the urine TFV assay kits resulted in variable availability of test kits across the visits and sites (Table S1).

Counselling staff were trained on how to conduct the urine TFV test, interpret results, and counselling about facilitators and barriers to PrEP adherence, which incorporated results from the POC urine TFV results when available, or followed standard PrEP adherence counselling guidelines if urine tests were not available. In either situation, challenges, barriers and facilitators to PrEP use were addressed using a client‐centred approach. An acceptability questionnaire about the urine TFV test was administered at month 6.

### 2.5 Ethical review

The study was approved by ethics review committees at each site. All participants provided written informed consent in English or their local language. Following local regulations, participants below the legal age of consent who were neither emancipated nor mature minors provided assent and informed consent was provided by their legal guardian.

### 2.6 Statistical analysis

Descriptive statistics were used to summarize baseline characteristics, self‐reported PrEP adherence since the prior visit and self‐reported PrEP side effects. PrEP uptake was defined as receiving a bottle at enrolment or a follow‐up visit. A positive POC urine TFV test, indicating ingestion of TDF/FTC PrEP in the prior 4−7 days, served as the objective marker of recent PrEP adherence. A substantial number of visits did not have urine tests (Table S1). Based on the assumption that missing test results were unrelated to participant characteristics, other than site and visit (Table S2), overall adherence was estimated using values from a logistic regression model of urine TFV test positivity predicted by site and visit.

Taking advantage of the “natural experiment” that occurred because urine testing was administratively not available for some participants at each visit, we assessed whether the use of the POC urine TFV test with tailored real‐time adherence counselling increased the frequency of positivity on the POC urine test at subsequent visits (e.g. recent PrEP adherence). Specifically, we assessed whether having experienced adherence counselling with the urine TFV test at month 1 or 3 (irrespective of the result of the test) was associated with a positive urine TFV test at a subsequent visit (e.g. month 3 or 6). This analysis included only participants who received PrEP at the prior visit, and was restricted to visits and sites with five or more participants who did and did not receive POC tests to reduce the risk of confounding by site. We used a modified Poisson regression model fitted according to generalized‐estimating‐equation methods to produce relative risk estimates [20]. Models were stratified by the study site. Age (<25 and ≥ 25 years) was considered a potential effect modifier given past HIV prevention trials in which adherence to product use was lower in young African women [21]. All analyses were conducted using SAS version 9.4.

## 3 RESULTS

### 3.1 Baseline characteristics

**Figure 1:**
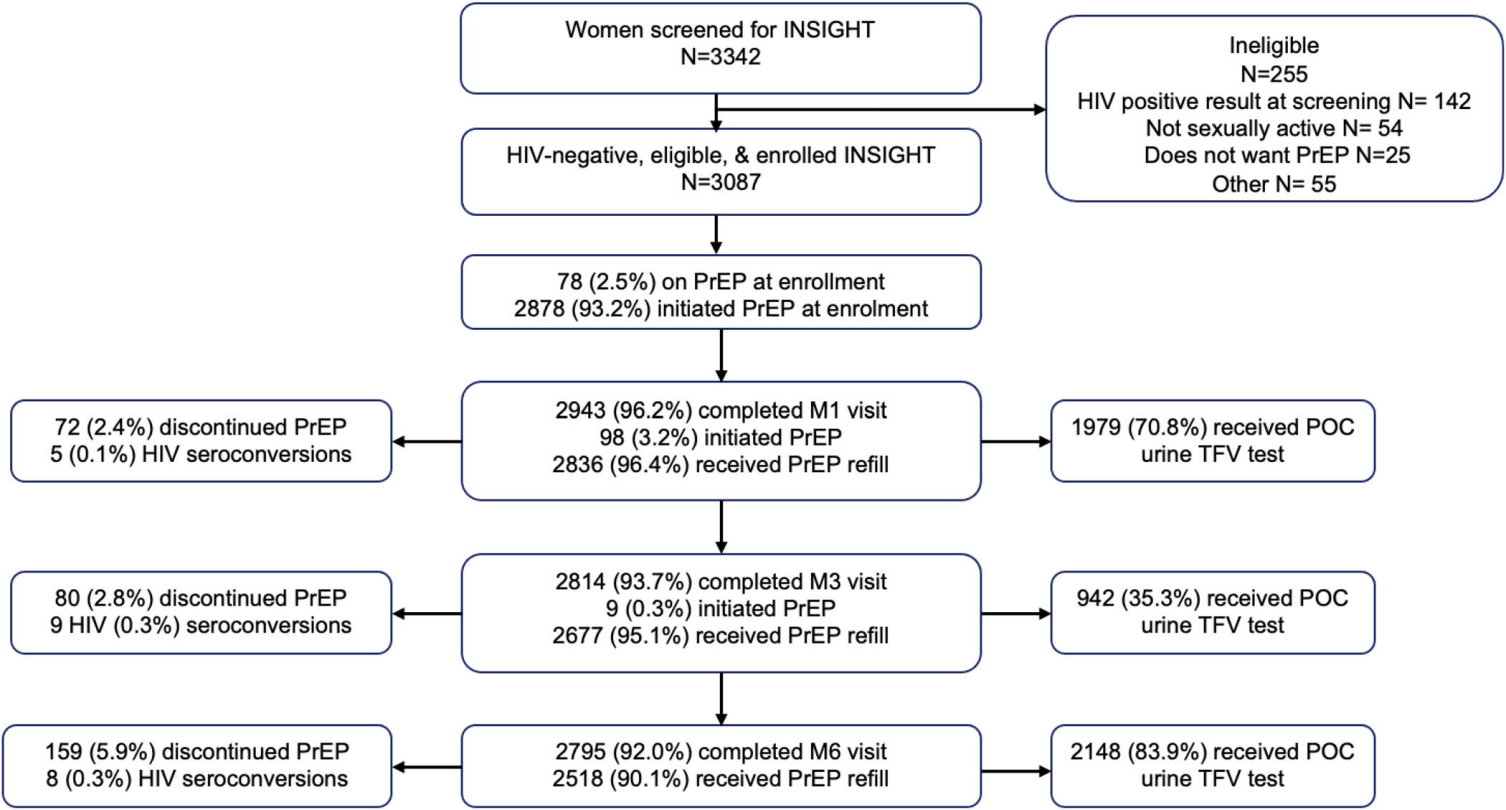
Study flow for INSIGHT cohort, depicts the number of participants who screened for the INSIGHT, reasons for ineligibility, enrolment, PrEP uptake at enrolment and follow‐up visits, refill dispensation, point‐of‐care urine tenofovir tests, HIV seroconversions and PrEP discontinuation at follow‐up visits.

A total of 3342 women screened for the INSIGHT cohort, of whom 3087 were eligible and enrolled into the prospective cohort study; 75.7% of participants were enrolled from South African sites and 24.3% from Eswatini, Kenya, Malawi, Uganda and Zambia. Reasons for ineligibility included living with HIV (*n* = 142), not sexually active in the prior 3 months (*n* = 54) and not interested in PrEP (*n* = 25) (Figure 1).

The median age of enrolled participants was 24 years (IQR 21−27) with 57.9% having completed a secondary education or higher; 61.2% were unemployed. Most (96.3%) participants reported a primary partnership, of whom 39.0% reported their partner had unknown HIV status, 1.5% reported that their partner was living with HIV and 29.5% reported that they thought their partner had other partners in the past 3 months. Overall, 72.5% of participants reported using one or more methods of contraception and injectable contraceptives was the most frequently reported method (39.7%). Three‐quarters of participants reported vaginal sex in the last 7 days and a similar proportion (74.7%) reported not using a condom with the last sex. The median modified VOICE risk score was five (IQR 4−7). Prior PrEP use was reported by 13.6% of participants and current PrEP use was reported by 2.5% at enrolment (Table 1).

**Table 1:** Baseline characteristics of adolescent girls and young women from East and Southern Africa participating in the INSIGHT cohort, 2022−2023

Baseline characteristics	Total *N* = 3087 Median (interquartile range, IQR) or *N* (%)
Age, years (median, IQR)	24 (21−27)
16−19	394 (12.8%)
20−25	1642 (53.2%)
26−29	1051 (34.0%)
Country	
South Africa	2337 (75.7%)
Other	750 (24.3%)
Education level	
No school or some primary	207 (6.7%)
Primary complete or some secondary	1093 (35.4%)
Secondary complete	1359 (44%)
Post‐secondary	428 (13.9%)
Relationship status	
Single/not partnered	115 (3.7%)
Partnered/not married	2709 (87.8%)
Married	263 (8.5%)
Primary partner characteristics	
Has primary partner	2972 (96.3%)
Age of primary partner, years	27 (24−30)
Primary partner is known to be living with HIV	46 (1.5%)
Primary partner of unknown HIV status	1159 (39.0%)
Has other sex partners in the past 3 months	912 (29.5%)
Recent sexual behaviour	
Vaginal sex in the last 7 days	2325 (75.4%)
Did not use condom last time had vaginal sex	2306 (74.7%)
Had anal sex in the past 3 months	139 (4.5%)
Occupation	
Unemployed	1889 (61.2%)
Student	538 (17.4%)
Informal sector (household help, parent caring for child)	291 (9.4%)
Employed formal sector	369 (12%)
Contraceptive use, not mutually exclusive	
None	849 (27.5%)
IUD	36 (1.2%)
Implant	494 (16.0%)
Injectable	1224 (39.7%)
Oral contraceptives	158 (5.1%)
Sterilization	4 (0.1%)
Other contraceptive method	591 (19.1%)
Used alcohol/substance use in the past 3 months	2034 (65.9%)
Experienced depressive symptoms in the prior past week^a^	1064 (34.5%)
Ever experienced intimate partner violence in lifetime	925 (36.6%)
Modified VOICE risk score, median (IQR)^b^	5 (4−7)
Past history of ever using pre‐exposure prophylaxis (PrEP)	419 (13.6%)
On PrEP at enrolment	78 (2.5%)

^a^ Includes women indicating they felt depressed between 1 and 7 days in last week on the Center for Epidemiologic Studies Depression Scale (CES‐D).

^b^ The modified VOICE risk score considers the following risk factors: age <25 years, married or cohabiting with a sexual partner, receiving financial or material support from a partner, primary male partner having other partners and alcohol use in the prior 3 months. A VOICE score of five or higher was associated with an annual HIV incidence of 6% in prior cohorts.

### 3.2 Retention and HIV seroconversion

Study retention was high with 96.2%, 93.7% and 92.0% of women attending follow‐up visits at months 1, 3 and 6, respectively. A total of 22 HIV seroconversions occurred through month 6 over 1590 person‐years of follow‐up for an HIV incidence of 1.38 per 100 person‐years (95% CI 0.97−2.08; Figure 1). Of the 22 women who acquired HIV during follow‐up, nine had a urine TFV result at the visit when they were first identified as having seroconverted, of which five (55.6%) tests were positive for urine TFV.

### 3.3 PrEP uptake, discontinuation and adherence

PrEP refills were received by 96.4% of participants attending study visits at month 1, 95.1% at month 3 and 90.1% at month 6 (Figure 1). Across all visits, 2230 (72.2%) of participants received a PrEP refill at each visit. Over 90% of participants reported that they felt protected while using PrEP, were less worried about getting HIV and felt “freer” when having sex (Table 2). The main challenges participants reported about PrEP use were forgetting to take pills (68.6%), forgetting to carry pills with them when travelling (42.7%) and feeling that they were not at risk for HIV (40.3%; Table 2). A total of 311 (10.1%) women discontinued PrEP during follow‐up and did not restart (Figure 1); common reasons for discontinuation were concerns about side effects (33.6%), not thinking they were at risk of HIV (11.0%), burdensome to take the tablet daily (8.1%) and peer influence (7.1%).

**Table 2:** Reported pre‐exposure prophylaxis (PrEP) side effects, disclosure, perceived impact and challenges

	Women enrolled in INSIGHT *N* = 3087
**PrEP use**	
PrEP initiated at enrolment	2878/3087 (93.2%)
PrEP initiated after enrolment	107/3087 (3.5%)
**PrEP experiences**	
PrEP side effects during INSIGHT	1902/3087 (61.6%)
PrEP disclosure during INSIGHT	2861/3087 (92.7%)
Unintended PrEP disclosure during INSIGHT	790/3087 (25.6%)
**PrEP impact on their lives (top three responses)**	
Feel protecting self	2946/3030 (97.2%)
Less worried about getting HIV	2752/3030 (90.8%)
Feel freer having sex	2750/3030 (90.8%)
**PrEP challenges (top three responses)**	
Forget to take pills	2078/3030 (68.6%)rep
Forget pills when travelling	1295/3030 (42.7%)
Do not feel at risk	1221/3030 (40.3%)

*Note*: The questions about HIV pre‐exposure prophylaxis (PrEP) side effects, disclosure, impact and challenges were asked at each visit. The responses about PrEP impact on their lives and challenges are from 3030 women at a follow‐up visit who had received PrEP at their prior visit and responded affirmatively to any PrEP impact or challenge.

Urine TFV tests were performed at month 1 for 1979 (70.8%) women who had initiated PrEP at enrolment and returned for the month 1 visit. For women who received a PrEP refill at month 1, 942 (35.3%) had a urine TFV test performed at month 3, and for the women who received PrEP refills at month 3, urine TFV testing was performed for 2148 (83.9%) at month 6. At month 3, four sites had no POC TFV testing due to manufacturer and importation delays (Table S1). Estimated recent PrEP adherence, based on observed urine TFV positivity adjusted for missing test results, was 72%, 71% and 65% at the month 1, 3 and 6 visits, respectively (Table 3). The characteristics that most significantly differentiated women who received a urine TFV test or not during follow‐up were site and visit (Table S2), reflecting supply issues in obtaining urine TFV tests.

**Table 3:** Pre‐exposure prophylaxis (PrEP) refills and adherence based on urine tenofovir positivity

	Month 1 *N* = 2943	Month 3 *N* = 2814	Month 6 *N* = 2795
PrEP refill received	2836 (96.4%)	2677 (95.1%)	2518 (90.1%)
Urine tests missing	964 (32.9%)	1872 (66.5%)	647 (23.1%)
Urine tests conducted	1979	942	2149
Urine TFV positive	1532	754	1417
Estimated TFV positivity rate^a^	72%	71%	65%

^a^ Estimated urine tenofovir (TFV) positivity rate is based on a prediction from a logistic regression model with predictors site and visit, to adjust for tests that were missing as a result of administrative delays (procurement and importation).

### 3.4 Effect of urine TFV testing and counselling on subsequent adherence

The analysis of the impact of adherence counselling with POC TFV testing at months 1 and 3 on PrEP adherence at subsequent visits included 1399 participants from 12 of the 20 sites. Of the 560 month 3 and 6 visits where the participant had not had a prior urine TFV test, 233 (41.6%) had a positive urine TFV test compared to 592 (70.6%) of the 839 visits where the participant had previously had a urine TFV test. Participants with prior experience with the urine TFV testing and real‐time counselling had a higher likelihood of having a subsequent positive POC TFV test compared to participants who did not have prior urine TFV testing; those having received one prior test had a 42% higher likelihood of a subsequent positive urine TFV test (adjusted OR = 1.42, 95% CI 1.27−1.60), and those having received two prior urine TFV tests had a 67% higher likelihood (adjusted OR = 1.67; 95% CI 1.41−1.98; Table 4). This effect was not modified by age (for women age <25 years, aOR = 1.5; for women ≥25, aOR = 1.4; interaction *p*‐value = 0.65).

**Table 4:** Association of urine tenofovir testing and counselling at month 1 or 3 as a predictor of urine tenofovir positivity at month 3 or 6

	Positive test result at visit (M3 and/or 6)	Risk ratio (95% confidence interval)	*p*‐value
Received testing at prior visit^a^			
Any prior urine test	641/782 (82%)	1.18 (1.1−1.26)	<.001
No prior urine test	509/769 (66.2%)	reference	
Number of prior urine tests^b^			
No prior test	233/560 (41.6%)	reference	
1 prior test	480/696 (69%)	1.42 (1.27−1.60)	<.001
2 prior tests	112/143 (78.3%)	1.67 (1.41−1.98)	<.001

*Note*: A modified Poisson regression model with generalized‐estimating‐equation methods was used to estimate relative risk estimates for whether having adherence counselling with the urine tenofovir test at month 1 or3 (irrespective of the result of the test) was associated with a positive urine TFV test at a subsequent visit (at month 3 or 6).

^a^ Eleven sites included to evaluate the association of any prior urine TFV test with a subsequent positive test: Emavundleni (Cape Town, SA), HSRC (Sweetwaters, SA), ICAP (Mbabane, eSwatini), Kamuzu (Blantyre, Malawi), MU‐JHU (Kampala, Uganda), Masiphumulele (Cape Town, SA), MatCH (Durban, SA), Ndevana (East London, SA), PHRU (Soweto, SA), SAMRC (Chatsworth, SA), Wits RHI Ward 21 (Johannesburg, SA).

^b^ Twelve sites included to evaluate the association of the number of prior urine TFV tests with a subsequent positive test: Aurum (Klerksdorp, SA), Aurum (Rustenburg, SA), CHRU (Johannesburg, SA), ICAP (Mbabane, eSwatinit), Kamuzu (Blantyre, Malawi), Madibeng (Brits, SA), Masiphumulele (Cape Town, SA), MatCH (Durban, SA), PHRU (Soweto, SA), SAMRC (Chatsworth, SA), Setshaba (Soshanguve, SA), Wits RHI Ward 21 (Johannesburg, SA).

Most women reported that the urine TFV test was helpful: 63.7% of 1999 women who received a positive result indicated that the positive test motivated them to take PrEP, 37.4% of 1204 women who received a negative test indicated that they were not surprised by the result, and 19.4% of 2887 women who received urine TFV testing and counselling reported that a counsellor helped them find ways to remember PrEP.

## 4 DISCUSSION

In this prospective multi‐site cohort of 3087 African AGYW, most of whom were PrEP‐naïve, PrEP uptake was very high (93.2% initially and 99.0% ever during the study). PrEP persistence was also very high with refills obtained at follow‐up visits by over 90% of women, and with 72.2% of women receiving PrEP refills throughout the 6 months of follow‐up. Interest in PrEP at the end of 6 months appeared high with 90.1% of women exiting the study with a PrEP refill and referral to public PrEP programmes. Recent PrEP adherence was relatively high based on a POC urine assay that detects TFV ingestion in the past 4 days, which indicated 72% adherence at month 1, declining slightly to 65% at month 6. Receiving one or two prior urine TFV tests with same‐day adherence counselling was associated with a 42% and 67% higher likelihood of a positive urine TFV result at a subsequent visit, respectively.

The almost universal acceptance of PrEP (99.0%) in the INSIGHT cohort is higher than the observed 90% in previous PrEP demonstration projects among African AGYW [2, 5]. This could reflect that some participants were recruited from a wait list for an efficacy trial of an investigational monthly PrEP drug, oral islatravir, for which enrolment was stopped in December 2021 due to a safety hold mandated by the US Food and Drug Administration [22, 23]. These participants may have had a high interest in novel oral PrEP agents and contributing to research efforts. Second, the COVID‐19 pandemic may have decreased access to PrEP through public clinics in Africa [24], creating more demand for PrEP access among those who found it through our study. Third, there is increasing awareness and acceptability of PrEP in communities since it was first introduced in African countries after the 2015 WHO recommendation and incorporated into national guidelines and programmes, including the PEPFAR DREAMS programme for AGYW. The high uptake was also associated with most women reporting perceived benefits of PrEP.

PrEP adherence at each visit was assessed by a novel POC urine assay which detects TFV ingestion in the prior week; recent adherence assessed via this objective metric was high with 72% having a positive result at month 1 which decreased modestly to 65% at month 6. This is encouraging and may reflect greater familiarity and acceptance of PrEP. A POC assay that provides an objective indicator of recent PrEP adherence may have greater utility than other measures as it may be easier for PrEP users to remember recent PrEP adherence behaviours in same‐day adherence counselling. Moreover, the test may facilitate more honest discussions about their recent PrEP use, as demonstrated in a separate study among Ugandan women [16], which may positively impact subsequent adherence. However, “white coat” or “pre‐appointment” dosing may account for some of the positive urine TFV results due to the social desirability to please the HIV prevention‐focused counsellors. In the open‐label extension of the iPrEX trial, white coat dosing was estimated to be 32% among men who have sex with men and transgender women. Among women randomized to the TDF/FTC arm and who acquired HIV during the HPTN 084 efficacy trial of injectable cabotegravir, white coat dosing as measured through comparisons of plasma TFV levels (which is a marker of use in the past 7 days) and intracellular TFV‐DP levels in dried blood spot (a marker of longer‐term PrEP use in the past 6−8 weeks) was estimated to be 25% among young women [25, 26]. In a recent study of young women on PrEP in Kenya who were aware that the urine TFV assay was being used as a measure of adherence, the urine TFV assay indicated 7% possible white coat dosing based on comparisons with tenofovir levels in hair (a marker of use in the prior 1−2 months); women who received the urine TFV assay were 3.5 fold less likely to have non‐adherence at month 12 based on detection of TFV in hair [15]. Acceptability of the urine TFV test and counselling was high with 93% reporting that they liked the urine TFV test and 64% of women who received a positive test indicating that the positive result motivated them to take PrEP.

The urine TFV assay provides rapid results, is simple to use, has a long shelf life and is anticipated to be low cost when commercially available, increasing its potential to be scalable for use in clinical settings for recent adherence monitoring and adherence counselling. In this setting, a counsellor should describe that the test detects their PrEP pill‐taking in the prior week and to focus the counselling on adherence behaviours in the past week, for which PrEP users should readily be able to remember their pill‐taking behaviour and reasons for missing doses. For those with a positive urine TFV result, the counsellor supported their recent use, asked about whether their PrEP use in the last week was typical and encouraged the client to take PrEP consistently. For those with a negative result, the counsellor provided supportive counselling about the challenges with daily pill‐taking and probed about whether their negative urine TFV assay indicates non‐use because of lack of perceived need, side effects, forgetting, or issues with storage, having PrEP if away from home, or fears of a partner or family member's reaction. Research is needed to assess the content and impact of counselling messages about recent PrEP adherence on subsequent longer‐term measures of PrEP adherence and persistence.

HIV incidence was 1.38 per 100 person‐years in this open‐label study of oral PrEP, which is modestly lower than the 1.85 per 100 person‐years observed among African AGYW in the TDF/FTC active comparator arm in the HPTN 084 efficacy trial of injectable cabotegravir in which 42% had plasma TFV levels indicating recent use, [27] and lower than the 2.41 per 100 person‐years in the FTC‐TDF arm in the PURPOSE 1 trial [28]. The observed HIV incidence is also lower than in oral PrEP demonstration projects among young African women which had lower adherence and persistence rates [2].

Limitations of this study include that although the study enrolled across 20 sites, 15 were in South Africa which enrolled 75% of participants, so generalizability is greatest for the South African context. Follow‐up was limited to 6 months, so longer‐term PrEP use was not assessed. Participants were not randomized to receive or not receive POC urine TFV testing and adherence counselling incorporating the results; an analysis indicated that the administrative cause of POC tests not being available by site and visit were most strongly associated with urine TFV testing and there was very little indication of confounding by participant characteristics. No evaluation was conducted of counsellors’ fidelity to counselling messages about the urine TFV results. Only nine of the 22 women who seroconverted to HIV had urine TFV results, limiting our ability to evaluate PrEP adherence in seroconverters. We were unable to quantify the degree to which white coat dosing may affect our estimates of PrEP adherence or the impact of the POC TFV test on subsequent adherence. Reimbursement for study visits and access to research staff and services through the study could have influenced participant motivation, retention and white coat dosing. Finally, the evaluation of urine TFV testing and counselling was nested within a prospective cohort of open‐label PrEP with trained research nurses and counsellors, many of whom had experience in PrEP counselling, and the results may not reflect implementation in programmatic and clinical settings.

## 5 CONCLUSIONS

PrEP uptake, adherence and persistence in the INSIGHT cohort were higher than observed in prior open‐label studies among African AGYW, as measured by an objective measure of recent adherence and PrEP refills through 6 months. These findings may reflect trends in growing population‐level acceptance and enthusiasm for PrEP, which could set the stage for faster introduction and scale‐up of novel PrEP products, and the introduction of which could build upon the knowledge and experiences of delivering oral PrEP. These findings also indicate that oral PrEP is an important and feasible option for many AGYW in the context of supportive adherence counselling and real‐time drug monitoring. Additional research is needed on how best to use the urine TFV test for real‐time PrEP counselling and to monitor PrEP adherence in order to optimize PrEP delivery and population‐level impact.

## COMPETING INTERESTS

BGM, MK, ZZ, EB, RP, CL, NM, PS, MS, LN, RC, MK, HN‐B, PK, KG, PM, AvH, SB, MJ, PdP, AW, RPHP, SD‐M, SP, RJ, JC and SM have nothing to disclose. SD‐M, RH and MG have received NIH research grants. DD and JV have served as a DSMB member on NIH‐funded grants and received NIH research grants. CC has received honoraria from Gilead and Merck as a scientific advisor, unrelated to this research, and research grants from NIH and BMGF.

## AUTHORS’ CONTRIBUTIONS

CC, RH, BGM and DD designed the research study. ZZ, EB, RP, CL, NM, PS, MS, LN, RCK, MK HN‐B, PK, KG, PMD, AvH, SB, MJ, PdP, RP, SD‐M, MK, JC, SM and RJ performed the research study. MG and GW contributed essential tools. SP and DD analysed the data. BGM, DD, RH, SP and CC wrote the paper. All authors have read and approved the final manuscript.

## FUNDING

The Bill and Melinda Gates Foundation provided funding for this study through investment INV‐004743.

## Supporting information




**Table S1**: Site‐specific rates of urine tenofovir tests conducted among women with PrEP refill from prior visit
**Table S2**: Individual participant characteristics as predictors of higher adherence and probability of receiving a urine tenofovir (TFV) test, among participants who had received PrEP at the prior visit and were included in the predictors analysis
**Table S3**: Site teams for the INSIGHT cohort

## Data Availability

The data that support the findings of this study are available from the corresponding author upon reasonable request through icrc@uw.edu, which will be reviewed by a central publications and manuscript committee.
