# Supplementary material for: High recent PrEP adherence with point‐of‐care urine tenofovir testing and adherence counselling among young African women: results from the INSIGHT cohort
Source: J Int AIDS Soc. 2024 Dec 9;27(12):e26389. doi: 10.1002/jia2.26389 (PMC11628190; doi:10.1002/jia2.26389)
Supplement: Supplementary file 1 — Table S1: Site‐specific rates of urine tenofovir tests conducted among women with PrEP refill from prior visit Table S2: Individual participant characteristics as predictors of higher adherence and probability of receiving a urine tenofovir (TFV) test, among participants who had received PrEP at the prior visit and were included in the predictors analysis Table S3: Site teams for the INSIGHT cohort [file JIA2-27-e26389-s001.docx]

**Supplemental Tables:**

**Table S1: Site-specific rates of urine tenofovir tests conducted among women with PrEP refill from prior visit**

|  | **Month 1** | | **Month 3** | | **Month 6** | |
| --- | --- | --- | --- | --- | --- | --- |
| **Site** | **Urine TFV test conducted N (%)** | **urine TFV results positive n/N (%)** | **Urine TFV test conducted N (%)** | **urine TFV results positive n/N (%)** | **Urine TFV test conducted N (%)** | **urine TFV results positive n/N (%)** |
| Mbabane, eSwatini | 81 (55.9%) | 52/81 (64.2%) | 58 (43%) | 43/58 (74.1%) | 122 (96.1%) | 51/122 (41.8%) |
| Kisumu, Kenya | 136 (95.1%) | 89/136 (65.4%) | 134 (95.7%) | 102/134 (76.1%) | 120 (99.2%) | 95/120 (79.2%) |
| Blantyre, Malawi | 93 (86.1%) | 70/93 (75.3%) | 30 (25.2%) | 16/30 (53.3%) | 99 (90%) | 68/99 (68.7%) |
| Masiphumulele, Cape Town, SA | 110 (76.4%) | 88/110 (80%) | 31 (21.8%) | 16/31 (51.6%) | 98 (73.1%) | 62/98 (63.3%) |
| Emavudleni, Cape Town, SA | 138 (92%) | 118/138 (85.5%) | 11 (8%) | 10/11 (90.9%) | 114 (94.2%) | 91/114 (79.8%) |
| Khayelitsha, Cape Town, SA | 113 (99.1%) | 98/113 (86.7%) | 140 (95.9%) | 118/140 (84.3%) | 129 (92.1%) | 105/129 (81.4%) |
| Durban (Chatsworth), SA | 128 (87.1%) | 100/128 (78.1%) | 39 (27.3%) | 28/39 (71.8%) | 111 (78.2%) | 76/111 (68.5%) |
| Durban (MATCH), SA | 123 (84.8%) | 88/123 (71.5%) | 64 (45.4%) | 51/64 (79.7%) | 112 (91.8%) | 86/112 (76.8%) |
| East London, SA | 139 (92.7%) | 133/139 (95.7%) | 78 (56.5%) | 75/78 (96.2%) | 97 (68.3%) | 86/97 (88.7%) |
| Wits Johannesburg, SA | 124 (67.4%) | 102/124 (82.3%) | 109 (58.9%) | 93/109 (85.3%) | 175 (97.2%) | 139/175 (79.4%) |
| Helen Joseph, Johannesburg, SA | 117 (79.6%) | 96/117 (82.1%) | 18 (13.6%) | 14/18 (77.8%) | 80 (73.4%) | 67/80 (83.8%) |
| Klerksdorp, SA | 31 (19.3%) | 17/31 (54.8%) | 1 (0.7%) | 0/1 (0%) | 98 (76.6%) | 30/98 (30.6%) |
| Ladysmith, SA | 136 (90.7%) | 89/136 (65.4%) | 0 (0%) | N/A | 0 (0%) | N/A |
| Madibeng, Brits, SA | 41 (29.7%) | 23/41 (56.1%) | 0 (0%) | N/A | 95 (76%) | 49/95 (51.6%) |
| Pietermaritzburg, SA | 147 (98.7%) | 122/147 (83%) | 71 (47.7%) | 66/71 (93%) | 143 (100%) | 120/143 (83.9%) |
| Pretoria, SA | 49 (32.7%) | 37/49 (75.5%) | 1 (0.7%) | 0/1 (0%) | 139 (97.9%) | 35/139 (25.2%) |
| Rustenburg, SA | 85 (50.6%) | 66/85 (77.6%) | 4 (2.7%) | 0/4 (0%) | 127 (94.1%) | 54/127 (42.5%) |
| Soweto, SA | 50 (33.8%) | 34/50 (68%) | 6 (4.5%) | 4/6 (66.7%) | 91 (68.4%) | 53/91 (58.2%) |
| Kampala, Uganda | 138 (93.9%) | 110/138 (79.7%) | 122 (90.4%) | 113/122 (92.6%) | 117 (89.3%) | 106/117 (90.6%) |
| Lusaka, Zambia | 0 (0%) | N/A | 25 (17.4%) | 5/25 (20%) | 82 (57.3%) | 44/82 (53.7%) |
| **Overall** | **1979 (68.8%)** | **1532/1979 (77.4%)** | **942 (33.2%)** | **754/942 (80%)** | **2149 (80.4%)** | **1417/2149 (65.9%)** |

**Table S2: Individual participant characteristics as predictors of higher adherence and probability of receiving a urine tenofovir (TFV) test, among participants who had received PrEP at the prior visit and were included in the predictors analysis**

|  | **Had a Urine TFV test** | |  | |
| --- | --- | --- | --- | --- |
|  | **Yes** | **No** | **Risk Ratio** | **P-value** |
| Age, years |  |  |  |  |
| 16-17 | 29 (50%) | 29 (50%) | reference |  |
| 18-21 | 396 (63.7%) | 226 (36.3%) | 1.03 (0.84, 1.27) | 0.745 |
| 22+ | 1030 (68.3%) | 478 (31.7%) | 1.09 (0.9, 1.34) | 0.377 |
| Education |  |  |  |  |
| No school or some primary | 49 (45.8%) | 58 (54.2%) | reference |  |
| Primary complete | 489 (66.7%) | 244 (33.3%) | 1.26 (1.04, 1.54) | 0.021 |
| Secondary complete | 685 (68.6%) | 314 (31.4%) | 1.24 (1.02, 1.52) | 0.034 |
| Post secondary | 232 (66.5%) | 117 (33.5%) | 1.2 (0.98, 1.48) | 0.081 |
| Alcohol drug use | 1019 (68.7%) | 464 (31.3%) | 0.96 (0.9, 1.03) | 0.279 |
| Depression | 620 (70.9%) | 255 (29.1%) | 1.09 (1.04, 1.15) | <.001 |
| Partner has multiple partners | 448 (70%) | 192 (30%) | 0.99 (0.94, 1.05) | 0.783 |
| Condom used last time had sex | 1054 (66%) | 543 (34%) | 0.94 (0.89, 0.99) | 0.025 |
| Past PrEP use | 179 (66.3%) | 91 (33.7%) | 1.03 (0.96, 1.11) | 0.424 |
| Visit |  |  |  |  |
| Month 3 | 331 (38.3%) | 534 (61.7%) | reference |  |
| Month 6 | 1124 (85%) | 199 (15%) | 2.28 (2.05, 2.53) | <.001 |
| Site |  |  |  |  |
| Aurum (Klerksdorp) | 98 (76.6%) | 30 (23.4%) | 0.7 (0.63, 0.78) | <.001 |
| Aurum (Rustenburg) | 127 (94.1%) | 8 (5.9%) | 0.84 (0.79, 0.9) | <.001 |
| CHRU (Johannesburg) | 80 (73.4%) | 29 (26.6%) | 0.66 (0.58, 0.74) | <.001 |
| ICAP (Mbabane) | 180 (68.7%) | 82 (31.3%) | 0.87 (0.79, 0.95) | 0.002 |
| Kamuzu (Blantyre) | 129 (56.3%) | 100 (43.7%) | 0.76 (0.67, 0.86) | <.001 |
| Madibeng (Brits) | 95 (76%) | 30 (24%) | 0.69 (0.62, 0.77) | <.001 |
| Masiphumulele (Cape Town) | 129 (46.7%) | 147 (53.3%) | 0.59 (0.53, 0.67) | <.001 |
| MatCH (Durban) | 64 (45.4%) | 77 (54.6%) | 1 (0.81, 1.24) | 0.964 |
| PHRU (Soweto) | 91 (68.4%) | 42 (31.6%) | 0.64 (0.56, 0.73) | <.001 |
| SAMRC (Chatsworth) | 39 (27.3%) | 104 (72.7%) | 0.56 (0.42, 0.74) | <.001 |
| Setshaba (Soshanguve) | 139 (97.9%) | 3 (2.1%) | 0.89 (0.85, 0.94) | <.001 |
| WITS RHI Ward 21 (Johannesburg) | 284 (77.8%) | 81 (22.2%) | reference |  |

**Table S3: Site teams for the INSIGHT cohort**

**International AIDS Care and Treatment Program, ICAP Eswatini Research Centre (Mbabane, Eswatini)**

Buyile Dlamini, Mabonga Dlamini, Mxolisi Dlamini, Temhlanga Dlamini, Samkelisiwe Pretty Dlamini-Zwane, Wandile Dube, Mthobisi Ginindza, Nontobeko Gwebu, Lomalondon Hlatshwako, Jessica Justman, Shandirai Kapuyanyika, Siphesihle Lukhele, Nomxolisi Mabuza, Nokuthula Mahlalela, Bongiwe P Malinga, Nothemba Masuku, Nkosingiphile Mashaba, Ayanda Monareng, Nomvula Motsa, Ritha Ncube-Sihlongonyane, Babongile Nkala, Nkosephayo Nkambule, Nomcebo Nkuna, Harriet Nuwagaba-Biribonwoha, Mfundo Nyembe, Ruben Sahabo, Nomsa Shongwe, Siphesihle Shongwe, Tholakele Shongwe, Sindy Nana Shongwe-Matse, Manqoba Sikhondze, Nontokozo Simelane, Thandazile Zikalala

**Kenya Medical Research Institute, KEMRI (Kisumu, Kenya)**

Loreta Achieng, Cynthia Pauline Aluoch, Daisy Eugene Anyango, Magdaline Asewe, Elizabeth Bukusi, Melvin Kiche, Elizabeth Koyo, Felix Mogaka, Vincent Momanyi, Alfred Obiero, Beatrice Obonyo, Lavine Awino Ochieng, Brenda Odera, Treezer Odhiambo, Josephine Odoyo, Alphonce Oguk, Victor Omollo, Brian Omondi, Pinkley Opiyo, Boblief Otieno, Eileen Otieno, Peris Otieno, Kevin Oware, Greshon Rota, John Bosco Tsetso

**Kamuzu Research Centre (Blantrye, Malawi)**

Effie Bello, Rachel Chihana, Lingstone Chiume, Sufia Dadabhai, Chikondi Dambula, Daniel Gondwe, Dumisile Huwa, Loveness Mwai Imaan, Melvin Kamanga, Desire Malichi, Moses Malunga, Lameck Manda, Josiah Mayani, Abgail Mnemba, William Mpute, James Msonda, Augustine Mwalwanda, Julliana Ngwira, Milika Nyirongo, Gloria Sakala, Juliet Seleman, Linly Seyama, Dean Soko, Mphatso Tandwe

**Setshaba (Soshanguve, South Africa)**

Lydia Sasabona Chauke, Nkgau Selaelo Chipape, Lizzie Dikeledi Ditabo, Mokhele Jacob Kaibe, Mokgadi Nelia Kubyana, Patience Faith Kwedza, Mamokoma Martina Madladla, Kedibone Baile Mahlangu, Tercia Terry Makhaphiedza, Morakane Alicia Makwela, Victoria Maleka, Nelly Nomthandazo Maphanga, Josephine Lindiwe Masemola, Hosea Malesela Matlebjane, Kagiso Elizabeth Mfomme, Kgaogelo Mirenda Mogane, Magdeline Kgaogelo Molapo, Katlego Eugine Morake, Edward Pule Moroke, Ronald Mololeng Moseri, Jane Matshidiso Motswaledi, Lebogang Letta Mpete, Elizabeth Khabonina Skhosana, Zinhle Zwane

**Qhakaza Mbokodo Research Centre (Ladysmith, South Africa)**

Nicole Gracie, Sandrah Hlatshwayo, Thabile Khumalo, Philippus Kotze, Nasima Lyons, Nomonde Mtshali, Michelle Nelson, Maria Otto, Fikile Rasimeni, Maryna Schoeman

**Masiphumulele (CapeTown, South Africa)**

Hlonipha Boqwana, Phakama Cofa, Rezeen Daniels, Keisha De Gouveia, Menna Duyver, Sinayo Dyubhele, Katherine Gill, Olwethu Kemele, Mellissa le Fevre, Anda Madikida, Owethu Mahali, Karabo Mahlangu, Nokupiwa Mahoyi, Thulisa Mayekiso, Sisipho Mehlo, Sanelisiwe Mbiko, Asisipho Mdayi, Bonga Mithi, Xolile Mhlanga, Ngosa Mulubwe, Babalwa Mnani, Sinothando Mqalo, Penelope P. Ngcobo, Thankdeka P. Nkosi, Buyiswa Ntsadu, Asiphe Ntshongontshi, Azola Olifant, Chido Ponde, Jade Roberts, Yamkelani Simakuhle, Nicola Stiekema, Nicola Thomas, Dorothy Zakariya, Ayabonga Zidlele

**Madibeng Centre for Research (Brits, South Africa)**

Marthie De Villiers, Carla Edeling, Emma Hlaletwa, Sonja Jordaan, Thabang Katees, Bongai Khatide, Keitumetse Kolobe, Julia Komane, Mary Kupa, Sally Lekalakala, Cheryl Louw, Tebogo Lusale, Letlhogonolo Maebana, Ralinah Maepa, Mamphi Mafoka, Mmathapelo Malefu, Martha Martins, Jane Mashigo, Rose Masilo, Lenah Masombuka, Oratile Matshaba, Karabo Matsila, Blessing Matsunyane, Boitumelo Milanzi, Valerie Mlotshwa, Thapelo Mmusi, Kefiloe Modikoe, Lerato Mokgethoa, Tebogo Molefe, Daphney Montoedi, Tsholofelo Mosito, Bernard Motau, Anna Mothata, Diana Motshweni, Minenhle Ndiweni, Qiniso Ndukuya, Cathy Neate, Lynette Ngubane, Sanny Nkentshani, Nomfundo Ntuli, Christina Phalali, Philemon Ramoshaba, Mamohau Sekonyana, Mavis Senne, Lorraine Shibambu, Nomonde Sibabela, Natasha Swart, Mmalerato Tsebe, Nkosinathi Tshabalala

**Helen Joseph Clinical HIV Research Unit, CHRU (Johannesburg, South Africa)**

Desiree van Amsterdam, Sharlaa Badal-Faesen, Jaclyn Bennet, Sephonono Molema, Noluthando Mwelase, Mohamed Rassool

**Emavundleni Research Centre (CapeTown, South Africa)**

Thobeka Coba, Thembakazi Dako, Nwabisa Danster, Pamela Dukwe, Llewellyn Fleurs, Gakiema Malan, Jenipher Gelant, Litha Gogo, Khadijah Gool, Janice Groenewald, Sibusiso Gumede, Xolani Gxako, Ridley Howard, Mustafaa Maarman, Pippa Macdonald, Nocwaka Magobiane, Ferial Mahed, Scott Hayden Mahoney, Pamela Phumla Makhamba, Nozicelo Mbiza, Eve Frances Mendel, Princess Hlengiwe Mkhize, Bukiwe Mngqebisa, Kgomotso Mocumi, Nombeko Cynthia Mpongo, Nobubele Mshudulu, Julio Muller, Priscilla Pamela Mvinjelwa, Ndiseka Nashwa, Theodorah Rirhandzu Ndzhukule, Nontshukumo Ngqabe, Nokubonga Londiwe Ntombela, Nazmie Pearce, Elaine Sebastian, Boneta Smitsdorff, Justine Stewart, Abigail Van der Linde

**Aurum Rustenburg Clinical Research Centre (Rustenburg, South Africa)**

Johanna Alida Baumgarten, Lerato Julia Block, William Lawwrence Brumskine, Emilia De Klerk, Engela Du Plooy, Megan Easton, Lebogang Isidora, Mabika Christian Kasongo, Sebaetsang Jeanette Kekana, Abigail Nomaswazi Kubeka, Mmarona Lekalakala, Sheiley Christina Lekotloane, Boitshoko Lekwe, Hellen Lukas, Octavia Makhosazana Madikwe, Moshukutjoane Lebogang Maila, Heeran Makkan, Perfect Tiisetso Malope, Omphile Petunia Masibi, Ellen Ditaba Matsane, Reabetswe Rorisang Kelly Moilwe, Caroline Mokhoko, Tshegofatso Dorah Moloatsi, Tsholofelo Mapula Mosito, Ireen Lesebang Mosweu, Seasebeng Pertunia Mothobi, Primrose Ketshepile Motswenyane, Phillip Mafori Mphahlele, Syiabonga Nhlapo, Joan Nyanhongo, Amukelani Dolly Nyathi, Themba Phakathi, Refilwe Gontse Powane, Tshegofatso Magnus Ramatlhape, Ni Ni Sein, Melissa Neo Senne, Bongiwe Stofile, Kgothatso Precious Tau, Mando Mmakhora Thaba, Lethabo Colleen Theko, Andrew Mojalefa Tlagadi, Joseph Molefi Tshabalala

**Perinatal HIV Research Unit-HIV Prevention Clinical Research Site, PHRU (Soweto, South Africa)**

Tseleng Daniel, Simphiwe Dlamini, Yolandie Fourie, Shamelle Govender, Nkosinathi Khasana, Christelle Kritzinger, Motlotlo Kwalane, Mazane Lembede, Catherine Lephoto, Abongile Lobi, Anita Marais, Mpho Motlamelle, Ravindre Panchia, Ayabonga Vika

**Human Sciences Research Council, Centre for Community Based Research, CCBR, Pietermaritzburg South Africa)**

Shannon Bosman, Pumla Dladla, Nontuthuko Dube, Lili-Marie Flax-Nel, Emmanuel Gabela, Alastair van Heerden, Thandanani Madonsela, Hlengiwe Mbokazi, Lenika Naiken, Ziphozenkosi Nzimande, Busisiwe Qumbisa, Mapule Ramorobi, Manchali Zwane

**MATCH Research Unit (Durban, South Africa)**

Mags Beksinska, Manjeetha Jaggernath, Yolandie Kriel, Nasiphi Magaqa, Noor Fathima Mahomed, Thandiwe Majola, Lindiwe Masaka, Mxolisi Mathenjwa, Mbali Mavundla, Nomvelo Mkhize, Sandelisiwe Mngomezulu, Nompumelelo Mnikathi, Amanda Mona, Sidelisiwe Msane, Unathi Ngcobo, Claudia Ngoloyi, Mita Ngwenya, Mosery Nzwakie, Bongeka Qiya, Hariska Ramlal, Nilayum Reddy, Jennifer Smit

**Foundation for Professional Development Ndevana Community Research Site (East London, South Africa)**

Pamela Bana, Joanne Batting, Leletu Buxeka, Nelesiwe Gantsho, Nolufefe Mazinyo, Chwayita Mgqamqo, Balindile Mlindazwe, Noxolo Ngoza, Nomazulu Notshokovu, Remco PH Peters, Drienie Pio, Zoliswa Siguca, Neliswa Swartbooi

**Wits Reproductive Health & HIV Research Unit Ward 21 Clinical Research Site (Johannesburg, South Africa)**

Joy Appolis, Oyetola Serah Babarinde, Nomakhaya Premrose Bojosi, Sinead Delany-Moretlwe, Nokuphiwa Dladla, Nelly Lebesi, Mosidi Calphurnia Maraba, Samukelo Vannessa Mbele, Nomfundo Dahlia Menzi, Thembekile Jemima Mokhele, Mapaseka Josephine Palesa Molefe, Nontokozo Happiness Ndlovu, Nicole Poovan, Mandla Qoshwa, Zacharias Nicos Taoushanis, Lerato Thulare

**UCT Khayelitsha Clinical Research Site (CapeTown, South Africa)**

Mohammed Baksh, Erica Boshoff, Phillip Du Preez, Nwabisa Gom, Nonceba Gobe, Raundine Hoffman, Francisco Lakay, Mandy Liao, Monica Magwayi, Nomvula Makade, Nobom Masimini, Bulelwa Mnyango, Jessica Ndegane, Nosipho Nombona, Mzamo Ntanja, Gadisi Nthambeleni, Denise Prinsloo, Lucien Smith, Sakhiwo Tunzi, Relebohile Tsekela, Rene Van der Venter, Phathuxolo Vinqishe, Amy Ward

**Aurum Klerksdorp Clinical Research Centre (Klerksdorp, South Africa)**

Heeran Makkan, Mgcini Moyo, Juanita Market, Pearl Selepe, Andrew Tlagadi

**SA Medical Research Council - Chatsworth Clinical Research Site (Chatsworth, South Africa)**

Lakshmi Baboolall, Akhona Bhengu, Thobile Bhengu, Mbongeleni Buthelezi, Tamon Cafun-Naidoo, Kerusha Chunderduri, Kumari Dewrance, Busisiwe Gumede, Vusi Hlambisa, Gugu Hlongwane, Ursula Jannsen, Nitesha Jeenarain, Bulelani Madonsela, Keshnee Maharajh, Gugulethu Majozi, Ayanda Mavundla, Mandisa Mchunu, Zinhle Miya, Sibongile Mkhungo, Lesiba Molekoa, Gerald Mphisa, Nondumiso Muthwa, Thabisile Mthembu, Logashvari Naidoo, Bukekile Ndlovu, Thuthukani Nkosi, Nokwazi Ntuli, Bridget Phakathi, Ashmintha Ramjeith, Mayuri Reddy, Qholokazi September, Sandisiwa Tyandela

**Makerere University/Johns Hopkins University (MU-JHU) (Kampala, Uganda)**

Juliet Asello, Biira Florence Asiimwe, Eleanor Nelly Birungi, Phionah Kibalama Bridget, Aida Bukirwa, Carolyne Cheptora, Richard Isabirye, Enid Kabugho, Judith Kainza, Hadijah Kalule, Betty Kamira, Ann Kankindi, Gladys Kasangaki. Annah Kebirungi, Doreen Kemigisha, Beatrice Kiiza, Nasif Kisambira, Annet Mirembe, Brenda Gati Mirembe, Dorcas Mirembe, Emmie Ouma Mulumba, Joselyne Nabisere, Christine Nagawa, Clemensia Nakabiito, Rita Nakalega, Catherine Nakaye, Teopista Nakyanzi, Justine Nakyeyune, Irene Nalumanya, Olive Victoria Namakula, Genevieve Nambalirwa, Maria Janine Nambusi, Agnes Namuddu, Rosemary Namwanje, Winnie Nansamba, Stella Nanyonga, Sophie Clare Nanziri, Bernadette Kirabo Nayiga, Miscah Babirye Otim, Ivan Rukundo, Imelda Maria Ssekamatte, Hassan Ssemere, Aisha Zalwango

**Kamwala Health Centre (Lusaka, Zambia)**

Chanda Mable Banda, Esther Banda Chabu, Beene Chembo, Godwin Chigande, Modesta Chileshe, Manze Chinyama, Cynthia Chuuzwe, Christine Claudine, Fredrick Kandeke, Margaret Kasaro, Dora Kasempa, Tina Malunga, Nelly Mwansa Mandona, Elizabeth Mubanga, Mayaba Joyela Mudenda, Dorothy Muyangwa, Godfridah Mwamba, Penelope Chama Mwamba, Humphrey Mwape, Fridah Ngwenya, Susan Phiri, Elina Selembe, Josephat Tembo, Laston Zulu, Violet Zulu
